# New Journal

**Published:** 1856-07

**Authors:** 


*New Journal.*

The *Western Journal of Medicine*, for many years published at Louisville, Ky., has been discontinued, and in its place we have before us Volume I, Number 1, of the *Louisville Review*, a bi-monthly journal of practical medicine and surgery, edited by S. D. Gross, M.D., Professor of Surgery in the University of Louisville, and T. G. Richardson, M.D., Demonstrator of Anatomy in the University of Louisville.

The *Review* contains 144 pages, is gotten up in the best style of the typographic art, and from the well-known talent of its editors will doubtless command an extensive patronage. The present number contains a lengthy review of Mansfield's Memoir on the Life and Services of the late Dr. Drake, accompanied by a good engraved likeness; a review of the Life and Times of Dr. August Gottlieb Richter; and of Dr. Budd on the Diseases of the Stomach. These are all ably-written and interesting papers. It is the design of the editors to give the review department of this journal greater prominence than is usual with the journals of this country.

